# Spatially distinct accretion of docosahexaenoic acid and eicosapentaenoic acid in a mouse model of Alzheimer's disease prior to plaque deposition

**DOI:** 10.1002/alz70855_107792

**Published:** 2025-12-25

**Authors:** Laura Beth Beth McIntire, Artur Lazarian, Nicholas Bartelo, Jason A Mares, Ana Paula Costa, William Dartora, Krista Minéia Wartchow, Jan Krumsiek, Tal Nuriel, Vilas Menon

**Affiliations:** ^1^ Weill Cornell Medical College, Brain Health Imaging Institute, Department of Radiology, New York, NY, USA; ^2^ Weill Cornell Medicine, Brain Health Imaging Institute, Department of Radiology, New York, NY, USA; ^3^ Weill Cornell Medicine, New York City, NY, USA; ^4^ Columbia University Irving Medical Center, New York, NY, USA; ^5^ Taub Institute for Research on Alzheimer's Disease and the Aging Brain, New York, NY, USA; ^6^ University of Pittsburgh, Pittsburgh, PA, USA; ^7^ Weill Cornell Medicine, New York, NY, USA; ^8^ Institute for Computational Biomedicine, Englander Institute for Precision Medicine, Department of Physiology and Biophysics, Weill Cornell Medicine, New York, NY, USA; ^9^ Department of Cell Biology and Pathology, New York, NY, USA; ^10^ Center for Translational & Computational Neuroimmunology, Columbia University Irving Medical Center, New York, NY, USA

## Abstract

**Background:**

Progressive dysregulation of polyunsaturated fatty acids such as docosahexaenoic acid is commonly observed in Alzheimer's disease (AD) and other neurodegenerative diseases. The culpability of dyshomeostasis of lipid metabolism in AD is further supported by the prevalence of lipid binding proteins in genome wide association studies for AD.

**Method:**

We acquired spatial lipidomic profiles in AD model mouse brain using the rapidly emerging technology, Desorption Electrospray Ionization imaging mass spectrometry (DESI‐IMS). Our studies are the first to identify a spatially distinct accretion of docosahexaenoic acid and eicosapentaenoic acid in the globus pallidus prior to plaque deposition. We found a differential spatial lipid profile during aging of wild type mice which was distinct from spatial lipidomic changes during aging in an AD mouse model. To quantify spatially distinct differences, we developed a novel pipeline specific for IMS spatial data.

**Result:**

Using spatial lipidomics, we identified the differential accretion of polyunsaturated fatty acids docosahexaenoic acid and eicosapentaenoic acid in the globus pallidus concurrent with soluble amyloid b‐peptide accumulation, but prior to plaque deposition. We further identified families of lipids which similar trajectories during aging in a wild type mouse model which were distinct from the AD mouse model.

**Conclusion:**

Novel lipid species and metabolic pathways are dysregulated during aging and in a mouse model of AD. Spatial lipidomic studies using DESI‐IMS have the potential to identify novel and regionally specific lipid biomarkers in normal aging and AD.

